# Potent Anti-HIV Activities and Mechanisms of Action of a Pine Cone Extract from *Pinus yunnanensis*

**DOI:** 10.3390/molecules17066916

**Published:** 2012-06-06

**Authors:** Xuan Zhang, Liu-Meng Yang, Guang-Ming Liu, Ya-Juan Liu, Chang-Bo Zheng, Yong-Jun Lv, Hao-Zhi Li, Yong-Tang Zheng

**Affiliations:** 1 Key Laboratory of Animal Models and Human Disease Mechanisms of the Chinese Academy of Science & Yunnan province, Kunming Institute of Zoology, Chinese Academy of Sciences, Kunming 650223, China; Email: snoopykm@126.com (X.Z.); lmyang@mail.kiz.ac.cn (L.-M.Y.); liushui0703@126.com(Y.-J.L.); 2 School of Pharmaceutical Science, Kunming Medical University, Kunming 650500, China; 3 Graduate School of the Chinese Academy of Science, Beijing 100039, China; 4 School of Pharmacy, Dali University, Dali 671000, China; Email: lgm888999@yahoo.com.cn (G.-M.L.); lvyongjun1939@126.com (Y.-J.L.); lihaozhi1943@126.com (H.-Z.L.); 5 School of Pharmacy, China Pharmaceutical University, Nanjing 210009, China; Email: normalbz@gmail.com

**Keywords:** *Pinus yunnanensis*, pine cone, anti-HIV activity, reverse transcriptase, fusion

## Abstract

The anti-HIV activities of a pine cone extract (YNS-PY-F) from *Pinus yunnanensis* have been evaluated, and its mechanisms of action were also explored. The pine cone extract, YNS-PY-F, potently inhibited HIV-1_IIIB_, HIV-1_RF_, HIV-1_A17_, HIV-1_AO18_ and HIV-2_ROD_ and induced cytopathic effect in C8166 cells with EC_50_ values of 0.96 μg/mL, 1.53 μg/mL, 0.88 μg/mL, 7.20 μg/mL and 6.17 μg/mL, respectively. The quantification of a p24 production assay showed that YNS-PY-F significantly inhibited the acute replication of HIV-1_IIIB_, HIV-1_RF_, HIV-1_A17_ and HIV-1_AO18_ in C8166 cells. An MTT assay showed that YNS-PY-F also significantly inhibited the HIV-1_IIIB_ induced cytolysis in MT-4 cells with an EC_50_ value of 2.22 μg/mL. The mechanism assays showed that YNS-PY-F had potent inhibitory effects on the fusion between infected cells and uninfected cells, and the activity of HIV-1 reverse transcriptase, with EC_50_ values of 7.60 μg/mL and 4.60 μg/mL, respectively. Overall, these data suggest that the pine cone extract from *Pinus yunnanensis* has potent inhibitory activities against HIV-1_IIIB_, HIV-1_RF_, RT inhibitor-resistant strains HIV-1_A17_ and HIV-1_AO18_, and HIV-2_ROD_, and its anti-HIV mechanisms include inhibition of HIV entry and inhibition of reverse transcriptase activity.

## 1. Introduction

Human immunodeficiency virus (HIV) is a lentivirus that causes acquired immunodeficiency syndrome (AIDS). UNAIDS estimated that there were some 34 million people (31.6 million–35.2 million) people living with HIV, including 2.7 million (2.4 million–2.9 million) new infections diagnosed at the end of 2010.

Antiretroviral therapy is still the major strategy for HIV infection. Considerable success has been achieved in the treatment of HIV infection, and more than two-dozen antiretroviral drugs are available targeting several distinct steps in the viral replication cycle. However, drug toxicity, drug resistance, adverse drug–drug interactions, and accompanying poor patient adherence are still the major factors leading to treatment failure [1]. Therefore, the discovery of novel drugs with new mechanisms of action, low toxicity, high activity and well tolerability is still a challenging issue in HIV treatment.

Medicinal plants play an important role in supporting healthcare system in the World. According to the World Health Organization (WHO), 80% of the rural population in developing countries mainly utilizes locally available medicinal plants for their primary healthcare needs. Nowadays, a large variety of natural products from medicinal plants, such as ribosome inactivating proteins, alkaloids, flavonoids, lignans, polysaccharides and so on, have been found to inhibit unique enzymes and proteins crucial to the life cycle of HIV, including the reverse transcription process, virus entry, the integrase or protease [2,3,4]. Therefore, screening potential anti-HIV agents from medicinal plants may be a rapid and effective way for drug discovery.

Pines are trees in the genus *Pinus*, in the family Pinaceae. There are about 115 species of pine, although different authorities accept anywhere between 105 and 125 species. Pine cones of some species of *Pinus* have been used for many years in the treatment of bronchitis, cough, asthma and other diseases in traditional Chinese medicine. Researchers have found that pine cone extracts or isolates from some species of *Pinus* in the Pinaceae family have antiviral, antitumor and immunopotentiating activities [5].

The significant anti-HIV activity of the pine cone extracts or isolates from *Pinus nigra* Arnold, *Pinus parvifloria* Sieb. et Zucc and *Pinus elliottii* var. Elliottii show the potential of pine cones as ideal therapeutic agents for the treatment of AIDS [6,7,8,9]. *Pinus yunnanensis* is also a member of the genus *Pinus* of the Pinaceae family, distributed mainly in the southwest of China [10]. However, there is still no report on whether the pine cone extract from *Pinus yunnanensis* has anti-HIV activities. In the present study, the anti-HIV activities of a pine cone extract (YNS-PY-F) from *Pinus yunnanensis* were evaluated, and its mechanisms of action were also explored.

## 2. Results and Discussion

### 2.1. Cytotoxities and Anti-HIV Activities

Drug efficacy and drug safety are two sides of the same coin, so they should be evaluated simultaneously [11]. In this study, the cytotoxities and anti-HIV activities *in vitro* were evaluated simultaneously. The cytotoxities against C8166 and MT-4 cells were evaluated by an MTT assay. In order to evaluate the anti-HIV activities of the pine cone extract (YNS-PY-F) from *Pinus yunnanensis*, cytopathic effect assay (syncytia assay), quantification of p24 production assay and MTT assay were used in this study.

**Figure 1:**
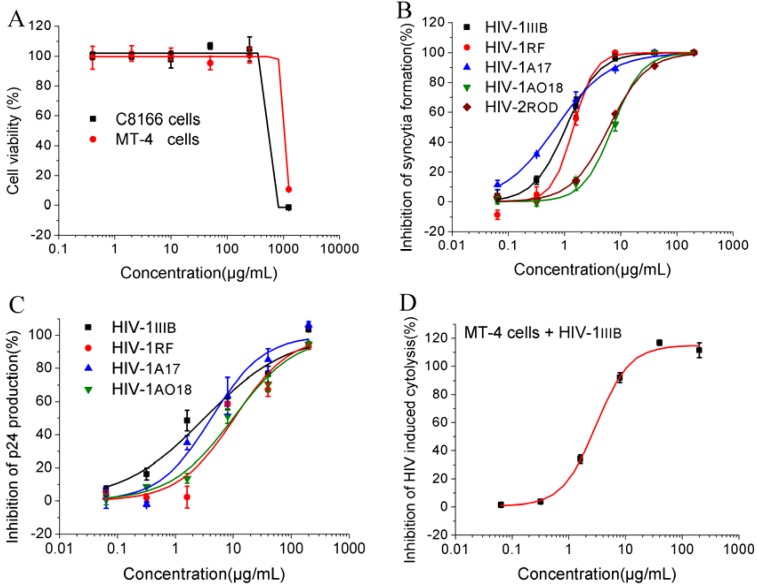
Cytotoxities and anti-HIV activities of YNS-PY-F. Cytotoxities on C8166 and MT-4 cells were measured by MTT assay (**A**); Inhibitory activities on HIV-1_IIIB_, HIV-1_RF_, HIV-1_A17_, HIV-1_AO18_ and HIV-2_ROD_ in C8166 cells were measured by syncytia reduction assay (**B**); Inhibitory activities on acute replication of HIV-1_IIIB_, HIV-1_RF_, HIV-1_A17_ and HIV-1_AO18_ in C8166 cells were measured by quantification of p24 antigen production (**C**); Inhibitory activity on HIV-1_IIIB_ induced cytolysis in MT-4 cells was measured by MTT assay (**D**). The data shown in the figure are a representative of at least two independent experiments.

The cytotoxities and anti-HIV activities of YNS-PY-F are summarized in Table 1. The cytotoxity assay showed that YNS-PY-F had low cytotoxities against C8166 and MT-4 cells, with CC_50_s of 438.73 μg/mL and 922.47 μg/mL, respectively (Table 1, Figure 1A). The syncytia assay showed that YNS-PY-F potently inhibited the cytopathic effect induced by different HIV strains, including laboratory-derived virus strains HIV-1_IIIB_ and HIV-1_RF_, RT nonnucleoside inhibitor-resistant strain HIV-1_A17_ and nucleoside inhibitor-resistant strains HIV-1_AO18_, and HIV-2_ROD_, with EC_50_s of 0.96 μg/mL, 1.53 μg/mL, 0.88 μg/mL, 7.20 μg/mL and 6.17 μg/mL, respectively, with selectivity indexes (SI = CC_50_/EC_50_) of 457.01, 286.75, 498.56, 60.93 and 71.11, respectively (Table 1, Figure 1B).

**Table 1:** Cytotoxities and anti-HIV activities of YNS-PY-F. CC_50_ is the concentration that inhibits 50% of cell viability. EC_50_ is the effective concentration that inhibits 50% of viral production or enzyme activity.

Samples	Cells		HIV strains/enzyme		Assay		CC_50_ (μg/mL)		EC_50_ (μg/mL)		SI	
YNS-PY-F	C8166		HIV-1_IIIB_		CPE		438.73		0.96		457.01	
					p24				3.98		110.23	
			HIV-1_RF_		CPE				1.53		286.75	
					p24				9.48		46.28	
			HIV-1_A17_		CPE				0.88		498.56	
					p24				4.04		108.60	
			HIV-1_AO18_		CPE				7.20		60.93	
					p24				9.79		44.81	
			HIV-2_ROD_		CPE				6.17		71.11	
			H9/HIV-1_IIIB_		CPE				7.60		57.73	
	MT-4		HIV-1_IIIB_		MTT		922.47		2.22		415.53	
	—		RT		ELISA		—		4.60		—	
AZT	C8166		HIV-1_IIIB_		CPE		1120.84		1.30 ^a^		862,184.62	
					p24				1.94 ^a^		577,752.58	
			HIV-1_RF_		CPE				2.95 ^a^		379,945.76	
					p24				6.48 ^a^		172,969.14	
			HIV-1_A17_		CPE				4.22 ^a^		265,601.90	
					p24				4.11 ^a^		272,710.46	
			HIV-2_ROD_		CPE				3.07 ^a^		365,094.46	
	MT-4		HIV-1_IIIB_		MTT		358.50		0.85 ^a^		421,764.71	
NVP	C8166		HIV-1_AO18_		CPE		—		61.70 ^b^		—
					p24		—		42.11 ^b^		—
T20	C8166		H9/HIV-1_IIIB_		CPE		—		14.96 ^a^		—
PFA	—		RT		ELISA		—		1.03		—

SI (selective index) = CC_50_/EC_50_. CPE: cytopathic effect. ^a^: ng/mL. ^b^: pM. The data shown in the table are means of at least two independent experiments.

The inhibitory activities of YNS-PY-F on HIV-1_IIIB_, HIV-1_RF_, HIV-1_A17_ and HIV-1_AO18_ were further confirmed by quantification of p24 antigen production with an ELISA method. Like in the syncytia assay, YNS-PY-F had potent inhibitory activities against p24 antigen production of the four HIV-1 strains in C8166 cells, with EC_50_s of 3.98 μg/mL, 9.48 μg/mL, 4.04 μg/mL and 9.79 μg/mL, respectively (Table 1, Figure 1C), suggesting that YNS-PY-F could inhibit the acute replication of these HIV-1 strains *in vitro*. The inhibitory activities on HIV-1_IIIB_ induced cytolysis in MT-4 cells was also assayed by MTT method, the result showed that YNS-PY-F potently inhibited the HIV-1_IIIB_ induced cytolysis in MT-4 cells with EC_50_ values of 2.22 μg/mL and SI of 415.53 (Table 1, Figure 1D), which was much higher than pine cone extracts from *Pinus parviflora* Sieb et Zucc. and *Pinus elliottii* var. Elliotti, with SI of 14 and 28, respectively [6].

The results showed that the pine cone extract from *Pinus yunnanensis* has significant antiviral activities against different HIV strains with a little different EC_50_ values. The different EC_50_ values may result from the different sensitivity of different viral strains to the pine cone extract. Interestingly, the EC_50_ values of YNS-PY-F against HIV-1_A17_ was significantly lower than HIV-1_AO18_, suggesting that YNS-PY-F has more potent antiviral activity against HIV-1_A17_ than HIV-1_AO18_, although the two viral strains are both RT inhibitor-resistant strains. This may be explained by their different mutation sites in the viral RT domain, as different mutation sites can lead to different sensitivity to drugs. HIV-1_A17_ is resistant to nonnucleoside RT inhibitors, while HIV-1_AO18_ is resistant to nucleoside RT inhibitors.

### 2.2. Inhibition on HIV-1 Fusion and Activities of Reverse Transcriptase

Given that YNS-PY-F had potent anti-HIV activities against different HIV strains, its anti-HIV mechanisms were further explored. The HIV entry process, including virus attachment and membrane fusion, is considered as an attractive target for chemotherapeutic intervention, as blocking HIV entry into its target cell leads to suppression of viral infectivity, replication and the cytotoxicity induced by virus-cell contacts [12]. Until now, threre are only two marketed HIV entry inhibitors, the fusion inhibitor enfuvirtide and the CCR5 antagonist maraviroc. HIV-1 reverse transcriptase is a well-known therapeutic target for treating HIV-1 infection and AIDS since there are no human equivalent enzymes and it is essential in HIV-1 infection and disease progression [13]. Although more than ten reverse transcriptase inhibitors have been approved by the U.S. Food and Drug Administration, the discovery of a new generation of HIV RT inhibitors is still urgent because of drug resistance. In the recent two years, a number of interesting, structurally diverse, small-sized compounds were found by virtual screening that may interact with HIV-1 reverse transcriptases [13,14,15].

Pine cones of different species of *Pinus* are known to be a rich resource of lignin-carbohydrate complexes (LCCs) and the major ingredient in hot water extracts of pine cones is LCC [6,8,9]. LCCs showed one order higher anti-HIV activity than tannins and flavonoids, and the anti-HIV activity induction mechanisms of LCCs include the inhibition of HIV adsorption to and penetration into the cells, and inhibition of reverse transcriptase and protease [16]. Pine cone of *Pinus yunnanensis* is also abundant in lignin-carbohydrate complexes, and the major component of the hot water extract of pine cone was also lignin-carbohydrate complex (LCC) [17]. Therefore, we further investigated whether YNS-PY-F, a hot water extract from *Pinus yunnanensis* can inhibit the fusion between normal cells and HIV-1 infected cells, and the activity of recombinant HIV-1 reverse transcriptase. The first fusion inhibitor enfuvirtide (T20) and the reverse transcriptase inhibitor foscarnet sodium (PFA) were used as positive control in fusion assay and reverse transcriptase activity assay, respectively.

**Figure 2:**
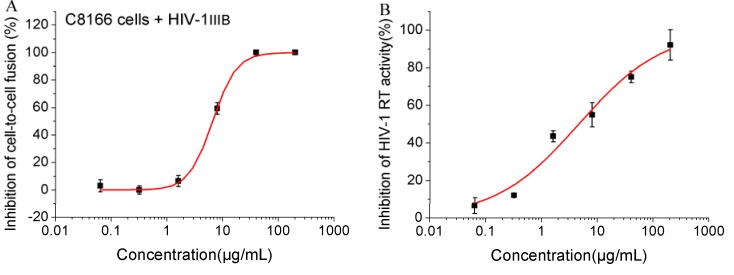
Inhibitory effects of YNS-PY-F on cell-to-cell fusion and RT activity. Inhibitory activity on the fusion of uninfected C8166 cells and H9 cells chronically infected HIV-1_IIIB_ was measured by syncytia reduction assay (**A**); Inhibitory activity on recombinant HIV-1 reverse transcriptase was measured by ELISA assay (**B**). The data shown in the figure are a representative of at least two independent experiments.

As we expected, the cell-to-cell fusion assay results showed that YNS-PY-F potently inhibited the fusion between normal C8166 cells and H9 cells chronically infected with HIV-1_IIIB_ with an EC_50_ of 7.60 μg/mL (Table 1, Figure 2A), suggesting that YNS-PY-F can inhibit HIV entry into the cells. The inhibition of recombinant HIV-1 reverse transcriptase activity was also detected by ELISA method using a commercial kit, the result showed that YNS-PY-F also significantly inhibited HIV-1 reverse transcriptase activity with an EC_50_ of 4.60 μg/mL (Table 1, Figure 2B), suggesting that it can be a potential reverse transcriptase inhibitor.

This extract showed both entry- and RT-inhibiting activity. However, it does not indicate that there exist multiple active compounds, as the major component was lignin-carbohydrate complex, and previous reports have proved that the mechanism of anti-HIV activity of LCC includes the inhibition of HIV adsorption to and penetration into the cells, and inhibition of reverse transcriptase [16]. Our results were consistent with the previous report.

Lignin-carbohydrate complexes have diverse pharmacological properties, such as antitumor, anti-microbial, antiparasite, antiviral and immunopotentiation actitity [16]. As the the major component of the pine cone extract was lignin-carbohydrate complex, so besides anti-HIV activity, the extract may have other various pharmacological properties. The potent anti-HIV activities and low cytotoxity *in vitro* indicate that the pine cone extract from *Pinus yunnanensis* has the potential to become an alternative medicine for HIV infection, however, its anti-HIV activities *in vivo* should be further evaluated.

### 2.3. Effect on HIV-1 Integrase Nuclear Translocation

Establishment of stable HIV-1 infection requires the efficient integration of the retroviral genome into the host DNA [18]. The integration event is mediated by the viral enzyme integrase (IN) [19,20]. IN bears a nuclear localization signal (NLS) domain located between residues 161 and 173. After reverse transcription, IN translocates to the nucleus in the form of a compact and stable preintegration complex (PIC), containing the viral reverse-transcribed genome and a number of virion-derived and cellular proteins, such as MA, Vpr and LEDGF/p75 [21]. The PIC nuclear import has been suggested to be mediated by the HIV-1 IN, via either the importin alpha or transportin 3 (TNPO3/transportin-SR2) pathways [22]. Therefore, HIV-1 IN nuclear translocation plays an important role in the life cycle of HIV-1 replication. Inhibition of IN nuclear translocation may lead to the failure of viral replication. A peptide bearing this sequence -NLS-IN peptide-inhibited nuclear accumulation of IN in transfected cell-cycle arrested cells [23]. D77, one benzoic acid derivative, has also been reported to inhibit IN nuclear translocation by disrupting the interaction between IN and LEDGF/p75 [24].

**Figure 3:**
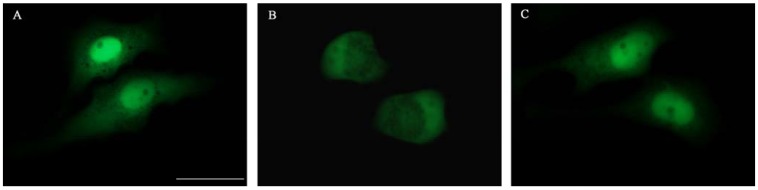
Effect of YNS-PY-F on the nuclear import of HIV-1 integrase in HeLa cells. (**A**) Negative control: EGFP-IN mainly distributed in the nucleus of HeLa cells; (**B**) D77 at 100 μg/mL: EGFP-IN mainly distributed in the cytoplasm of HeLa cells; (**C**) YNS-PY-F at 200 μg/mL: EGFP-IN mainly distributed in the nucleus of HeLa cells. The scale bar was 25 μm.

To investigate whether YNS-PY-F has inhibitory effect on the HIV-1 IN nuclear translocation, a cell based imaging screening method was used, and D77 was used as a positive control in this assay. However, the result showed that YNS-PY-F had no effect on HIV-1 integrase nuclear translocation even at 200 μg/mL. Similar to the negative control (cells treated with no drugs) (Figure 3A), EGFP-IN was mainly localized in nucleus of the HeLa cells treated with YNS-PY-F at 200 μg/mL (Figure 3C), while EGFP-IN was mainly localized in cytoplasm of cells treated with the positive control, D77 at 100 μg/mL (Figure 3B).

### 2.4. Effect on SDF-1α Induced CXCR4 Internalization

The chemokine receptors CXCR4 and CCR5, expressing respectively on human T cells and macrophages, are the two major coreceptors for HIV entry. Numerous efforts have been made to develop a new class of anti-HIV agents that target these coreceptors as an additional or alternative therapy to standard HAART [25]. There are two kinds of HIV entry inhibitors at present: CCR5 antagonists and HIV fusion inhibitors. Although many CXCR4 antagonists have also been studied, they are still at preclinical stages or have been suspended during development. Therefore, research of novel HIV entry inhibitors focuses on CXCR4 antagonists and attachment process inhibitors.

Given that YNS-PY-F potently inhibit the fusion of uninfected C8166 cells and H9 cells chronically infected HIV-1_IIIB_, and the cell lines used in anti-HIV activities tests were T cell lines, we further investigated its effect on CXCR4 by a cell based fluorescence imaging assay. SDF-1 and CXCR4 are believed to be a relatively “monogamous” ligand-receptor pair [26], therefore we used SDF-1α to induce CXCR4 internalization.

**Figure 4:**
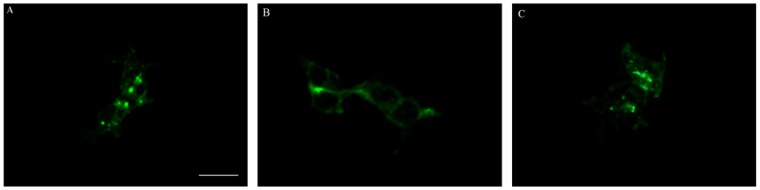
Effect of YNS-PY-F on SDF-1α induced CXCR4 internalization. (**A**) Negative control: EGFP-CXCR4 mainly distributed in the cytoplasm of 293 T cells treated by SDF-1α; (**B**) AMD3100 at 10 μM: EGFP-CXCR4 still mainly distributed in the cell membrane of cells treated by SDF-1α and AMD3100; (**C**) YNS-PY-F at 200 μg/mL: EGFP-CXCR4 mainly distributed in the cytoplasm of 293 T cells treated by SDF-1α andYNS-PY-F. The scale bar was 25 μm.

EGFP-CXCR4 was transported to cytoplasm of 293T cells after incubated with SDF-1α, a natural ligand of CXCR4 (Figure 4A), the positive compound AMD3100 significantly blocked SDF-1α induced CXCR4 internalization, EGFP-CXCR4 still distributed mainly in the membranes of 293T cells (Figure 4B), while EGFP-CXCR4 distributed mainly in cytoplasm of 293T cells treated with YNS-PY-F at 200 μg/mL (Figure 4C). The result showed that YNS-PY-F can not inhibit SDF-1α induced CXCR4 internalization, suggesting it can not be as a CXCR4 antagonist.

## 3. Experimental

### 3.1. Plant Materials and Extraction Procedure

**Figure 5:**
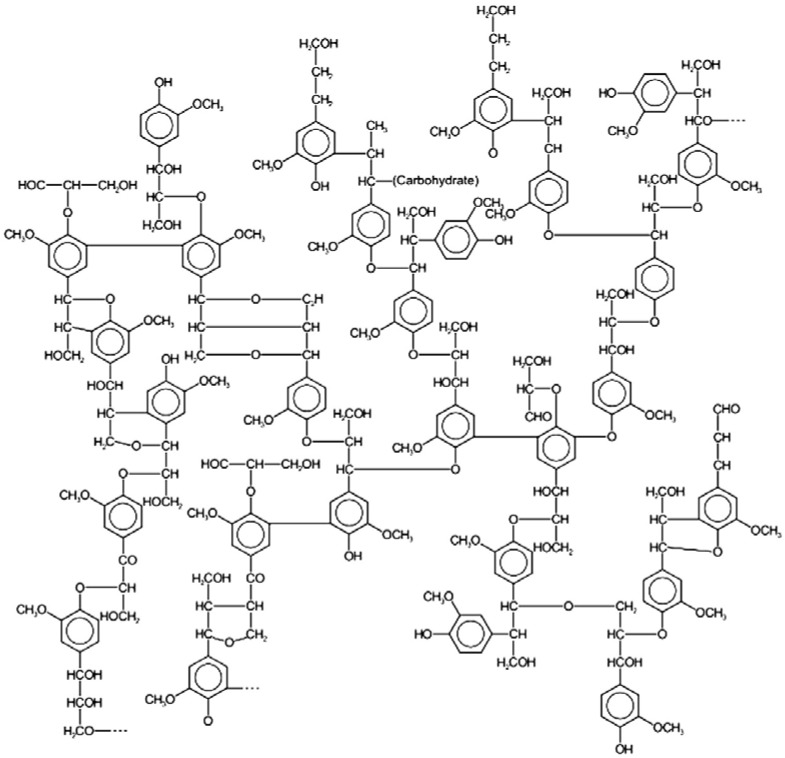
The structure of lignin-carbohydrate complex [16]. Carbohydrate represents polysaccharide portion of lignin–carbohydrate complex.

The air-dried pine cones of *Pinus yunnanensis* were collected in Yangbi County, Dali Prefecture of Yunnan Province, China, in April 2009. The plant material was identified by Xiao-Kuang Ma (School of Pharmacy, Dali University, Dali, China). A voucher specimen (BBP2010012PY) was deposited in School of Pharmacy, Dali University. The air-dried and powdered pine cones of *Pinus yunnanensis* (10.0 kg) were soaked in 95% ethanol solution (25 L) for 24 hours for three cycles, and then were filtered. The residues were extracted by boiling water for 2 hours for three cycles and then were extracted by 1% NaOH at room temperature, and finally filtered. The filtrate was adjusted to acidity by acetic acid, and then was filtered. The filtrate was precipitated by a threefold amount of ethanol solution, filtered, the filtrate was further precipitated by a fivefold amount of ethanol solution, and filtered, the sediments were washed sequentially with dehydrated ethanol and ethyl ether, then were evaporated under reduced pressure at 40 °C, finally 2.06 g of extract was obtained and coded as YNS-PY-F. The major ingredient in the hot water extract of pine cones from cones of *Pinus yunnanensis* was lignin-carbohydrate complex (LCC) [17].The structure of lignin-carbohydrate complex is shown in Figure 5.

### 3.2. Reagents and Chemicals

MTT, AZT (3'-azido-3'-deoxythymidine) and AMD3100 were purchased from Sigma. Horseradish peroxidase (HRP) labeled goat anti-human IgG was purchased from Dingguo Biotechnology Company (Beijing, China). SDF-1α was purchased from R & D Systems (Minneapolis, MN, USA). The p5F1 and monoclonal antibody (McAb) against HIV-1 p24 were prepared by our laboratory [27]. Enfuvirtide (T20) and Reverse Transcriptase Assay kit were purchased from Roche (Basel, Switzerland). Nevirapine (NVP) was purchased from ChemPacific (Baltimore, MD, USA). D77 was kindly donated by Shen Xu (Shanghai Institute of Materia Medica, Chinese Academy of Sciences, Shanghai, China).

### 3.3. Plasmid, Cells and Viruses

The pTRE2hyg-EGFP-C-IN plasmid and 293T stable cell line expressing CXCR4-GFP were kindly presented by David Chi Cheong (The Chinese University of Hong Kong, Hong Kong, China). Cell lines, including C8166 and MT-4 cells were maintained in Gibco complete RPMI-1640 (with 10% heat-inactivated FBS). HeLa and 293T cells were maintained in DMEM (with 10% heat-inactivated FBS). The laboratory-derived virus strains HIV-1_IIIB_ and HIV-1_RF_, RT nonnucleoside inhibitors resistant strain HIV-1_A17_ and nucleoside inhibitors resistant strain HIV-1_AO18_, and HIV-2_ROD_ were obtained from the NIH AIDS Research and Reference Reagent Program (USA). The 50% HIV tissue culture infectious dose (TCID_50_) was determined and calculated according to the method of Reed and Muench. All the viruses were stored in small aliquots at −70 °C.

### 3.4. Cytotoxicity Assay

MTT was used to assess the cytotoxicity of YNS-PY-F on C8166 cells and MT-4 cells [28]. Briefly, cells were seeded in a 96-well plate (4 × 10^4^ per well) in the absence or presence of various gradient concentrations of YNS-PY-F in triplicate, and then were incubated for 3 days (C8166) or 7 days (MT-4) at 37 °C, in a 5% CO_2_-humidified incubator. Then MTT (5 mg/mL in PBS) was added to each well. After incubating for 4 hours, 100 μL of 50% DMF/20% SDS was added, and the plate was incubated at 37 °C overnight. AZT was used as a positive control. The plate was read on a Bio-Tek Elx 800 ELISA reader at 595/630 nm. The 50% cytotoxic concentration (CC_50_) was calculated.

### 3.5. Inhibition of YNS-PY-F on HIV Induced Cytopathic Effects (CPE)

The inhibitory effect of YNS-PY-F on HIV induced cytopathic effect was examined by counting the number of syncytia under an inverted microscope [29]. Briefly, C8166 cells (4 × 10^4^ cells per well), infected with virus HIV-1_IIIB_, HIV-1_RF_, HIV-1_A17_, HIV-1_AO18_ and HIV-2_ROD_ at a multiplicity of infection (M.O.I. = 0.15), were seeded on 96-well plate in the absence or presence of various gradient concentrations of YNS-PY-F in triplicate. The final volume per well was 200 μL. After 3 days of culture, the cytopathic effect (CPE) was measured by counting the number of syncytia. AZT was used as a positive control. 50% effective concentration (EC_50_) was calculated.

### 3.6. Effect on HIV-1 Replication in Acute Infection

The inhibitory effect of YNS-PY-F on HIV-1 replication in acute infection was further examined by quantification of p24 production by ELISA as previously described [30]. Briefly, C8166 cells were infected with HIV-1_IIIB_, HIV-1_RF_ and HIV-1_A17_ and HIV-1_Ao18_ (M.O.I. = 0.15) at 37 °C, in a 5% CO_2_ humidified incubator for 2 hours to allow for viral adsorption. Cells were washed 3 times with PBS. 100 μL of cells (4 × 10^4^ cells) were seeded with 100 μL of various gradient concentrations of YNS-PY-F and incubated at 37 °C, in a 5% CO_2_-humidified incubator for 4 days. Then, 90 μL of supertanants were collected from each well and mixed with 10 μL of 5% Triton X-100. HIV-1 p24 expression was assayed by ELISA. AZT was used as a positive control.

### 3.7. Effect on HIV-1_IIIB_ Induced Cytolysis in MT-4 Cells

The inhibition of HIV-1 induced lytic effects in MT-4 cells was described previously [31]. Briefly, uninfected or HIV-1_IIIB_ infected (M.O.I. = 0.15) MT-4 cells (4 × 10^4^ cells per well) were seeded in 96-well plates with 100 μL of various gradient concentrations of YNS-PY-F. Plates were incubated for 7 days at 37 °C in a 5% CO_2_-humidified incubator. On day 7, the viability of cells was assessed by MTT as described above. AZT was used as a positive control.

### 3.8. Effect on Cell-To-Cell Fusion

Cell-to-cell fusion between normal C8166 cells and H9 cells chronically infected with HIV-1_IIIB_ was quantified under an inverted microscope as previously described [32]. Briefly, C8166 cells (3 × 10^4^) were co-cultured with H9 cells chronically infected with HIV-1_IIIB_ (1 × 10^4^) in the presence or absence of YNS-PY-F with various gradient concentrations. After incubation at 37 °C in a 5% CO_2_-humidified incubator for 6 hours, the number of syncytia was counted under an inverted microscope. Enfuvirtide (T20) was used as a positive control.

### 3.9. Effect on Recombinant HIV-1 Reverse Transcriptase (RT) Activity

HIV-1 reverse transcriptase (RT) activity was measured by ELISA RT kit using a commercially available kit (Roche) in accordance with the manufacturer’s instruction. Briefly, the recombinant HIV-RT solution, YNS-PY-F diluted in lysis buffer and the reaction mixture were sequentially added to each well in microplate modules, then were incubated for 2 hours at 37 °C. Then anti-DIG-POD solution was added, followed by substrate ABTS. Foscarnet sodium (PFA) was used as a positive control. The absorbance at 405 nm/490 nm (A405/490) was measured using a microplate reader (ELISA, Bio-Tek ELx 800, Winooski, VT, USA). The resulting signal intensity is directly proportional to the RT activity [33].

### 3.10. Effect on HIV-1 Integrase (IN) Nuclear Translocation

HeLa cells were cultured and maintained in Dulbecco’s modified Eagle’s medium (DMEM) supplemented with 10% fetal bovine serum (FBS), 100 μg/mL G418. 100 U/mL of streptomycin–penicillin (Invitrogen) at 37 °C in a 5% CO2 incubator. 24 hours before transfection, HeLa cells (4 × 105/well) were seeded onto a clear 24-well plate in DMEM containing 10% FBS. EGFP-C-IN expression vector was transfected into HeLa cells using Lipofectamine 2000 reagent (Invitrogen, Carlsbad, NM, USA) in accordance with the manufacturer’s instructions. The medium was removed 5 hours after transfection. Fresh medium containing pine cone extract solution was added at final concentration of 200 μg/mL. 24 hours after transfection, cells were fixed with 4% paraformaldehyde in PBS at room temperature for 15 minutes. Cell imaging was performed by Leica DMI4000 microscopy. D77 was used as a positive control [24].

### 3.11. Effect on SDF-1α Induced CXCR4 Internalization

293T stable cell line expressing CXCR4-GFP was cultured and maintained in Dulbecco’s Modified Eagle Medium supplemented with 10% fetal bovine serum (FBS), 25 mM HEPES and 1% of streptomycin-penicillin. Exponentially growing cells (1 × 105 cells/well) were seeded into a clear 96 well plate and cultured for 24 hours. Then the culture medium was removed and fresh culture medium containing pine cone extract solution was added at final concentration of 200 μg/mL. The pine cone extract solution was incubated with cells for 20 minutes, then SDF-1α, the natural ligand of CXCR4, was added at a final concentration of 10 nM, and incubated for 40 minutes. Fluorescent imaging was carried out to monitor CXCR4-GFP expression and internalization. Images were collected using Leica DMI4000 fluorescence microscope. AMD3100 was used as a positive control.

## 4. Conclusions

To our knowledge, our data demonstrated for the first time that a pine cone extract from *Pinus yunnanensis* has potent inhibitory activities against laboratory-derived virus strains HIV-1_IIIB_ and HIV-1_RF_, RT nonnucleoside inhibitors resistant strain HIV-1_A17_ and nucleoside inhibitors resistant strain HIV-1_AO18_, and HIV-2_ROD_, and the anti-HIV mechanisms include inhibition of HIV entry and inhibition of reverse transcriptase. The potent anti-HIV activities and low cytotoxity *in vitro* indicate that the pine cone extract from *Pinus yunnanensis* has the potential to become an alternative medicine for HIV infection, however, its anti-HIV activities and toxicity *in vivo* should be further evaluated.
